# The dynamics of explore–exploit decisions reveal a signal-to-noise mechanism for random exploration

**DOI:** 10.1038/s41598-021-82530-8

**Published:** 2021-02-04

**Authors:** Samuel F. Feng, Siyu Wang, Sylvia Zarnescu, Robert C. Wilson

**Affiliations:** 1 Department of Mathematics, Khalifa University of Science and Technology, Abu Dhabi, UAE; 2 Department of Psychology, University of Arizona, Tucson, AZ USA; 3 Cognitive Science Program, University of Arizona, Tucson, AZ USA; 4 Khalifa University Centre for Biotechnology, Khalifa University of Science and Technology, Abu Dhabi, UAE

**Keywords:** Cognitive neuroscience, Decision, Human behaviour

## Abstract

Growing evidence suggests that behavioral variability plays a critical role in how humans manage the tradeoff between exploration and exploitation. In these decisions a little variability can help us to overcome the desire to exploit known rewards by encouraging us to randomly explore something else. Here we investigate how such ‘random exploration’ could be controlled using a drift-diffusion model of the explore–exploit choice. In this model, variability is controlled by either the signal-to-noise ratio with which reward is encoded (the ‘drift rate’), or the amount of information required before a decision is made (the ‘threshold’). By fitting this model to behavior, we find that while, statistically, both drift and threshold change when people randomly explore, numerically, the change in drift rate has by far the largest effect. This suggests that random exploration is primarily driven by changes in the signal-to-noise ratio with which reward information is represented in the brain.

## Introduction

When choosing a class in college, should you exploit the Math class you are sure to ace, or explore the Photography class you know nothing about? Exploiting Math may be the way to a better grade, but exploring Photography—and finding that it scratches an itch you never knew you had—could be the path to a better life. As with all such ‘explore–exploit’ decisions, picking the optimal class is hard—explore too much and you’ll never finish your degree, exploit too much and, like us, you will do Math for the rest of your life.

From a computational perspective, the difficulty of explore–exploit decisions arises due to uncertainty about the outcome of each choice (Will I like photography or won’t I?) and the long time horizon over which the consequences of a choice can play out (If I like photography should I change my major?). To make an ‘optimal decision,’ that is a decision that maximizes our expected future reward, we need to average over all possible futures out to some time horizon^1^. However, averaging over all possible futures requires us to mentally simulate all possible futures—a computation that scales badly with uncertainty and horizon, and that is surely beyond what any brain can perform. Thus it is necessary for humans and animals to use heuristics and approximations to make practical explore–exploit choices that may be suboptimal in theory, but good enough in practice.

Inspired by research in machine learning (see^2^ for review), recent findings in psychology suggest that humans use two strategies to make explore–exploit decisions: an explicit bias for information (‘directed exploration’), and the randomization of choice (‘random exploration’)^3–13^. In directed exploration, a decision is made by comparing the expected values of exploring and exploiting. These expected values combine the predicted short-term payoff from picking an option once, the ‘expected reward,’ with an estimate of the long-term value of the information obtained from choosing that option, the ‘information bonus,’ also known as the future expected value^14^. The information bonus increases the value of exploratory options such that, when all else is equal, a directed explorer will always explore. In random exploration, the tendency to exploit the option with highest short-term expected reward is countered by ‘noise’ in the decision process. This noise introduces random variability to the decision, which sometimes leads to exploration by chance.

A key feature of both types of exploration is that they appear to be subject to cognitive control. Thus, when it is more valuable to explore—because there is more time to explore^4^, because the options are more uncertain^5,9^, or because exploring is the only way to gain information^10^—people exhibit more information seeking (directed exploration) and more variability in their behavior (random exploration). Exactly how the brain achieves this control of directed and random exploration is unknown.

**Figure 1:**
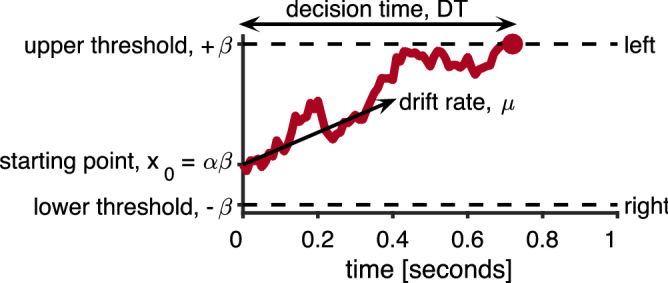
Schematic of the drift diffusion model showing the parameterization used in this paper.

In this work we develop a value-based drift-diffusion model^15–18^ of explore–exploit behavior to investigate how random exploration could be controlled. In this model, we assume that the decision between exploration and exploitation is accomplished by accumulating evidence over time. At any moment, we assume that this evidence is made up of a signal, $\mu$, proportional to the difference in expected values between the two options, and noise with variance $c^2$. The accumulation process starts from a starting point, $x_0$, that captures the initial bias towards one option or the other, and makes a decision when the accumulated evidence crosses a threshold at $+\beta$ for (e.g.) exploration and $-\beta$ for exploitation (Fig. 1).

In the drift-diffusion model, behavioral variability can be controlled by three different parameters corresponding to three different mechanisms for random exploration: (1) the signal, $\mu$, (2) the variance of the noise, $c^2$, or (3) the threshold, $\beta$. As shown in the “Results” section, at the level of choice it is impossible to distinguish between these three parameters. That is, identical changes in choice variability can be caused by changes in either signal, noise, or threshold (or a combination of all three). Thus, from choice data alone (which has been the main focus of the explore–exploit literature up to now), it is impossible to determine which of these processes controls random exploration.

In contrast to choices alone, it is possible to separate two of these processes (although unfortunately not all three^19^) using response times. In particular, we can distinguish a change in threshold from a change in the *ratio* of the signal to the noise. However, we cannot go further and attribute a signal-to-noise ratio (SNR) change to a change in signal, noise or both. For this reason, it is common to fix the variance of the noise, $c^2 = 1$, and interpret the drift parameter as a signal-to-noise ratio.

In this work, we fit a drift-diffusion model to choices and response times in a popular explore–exploit task (the Horizon Task^4^). Using this approach we find evidence that random exploration is primarily driven by changes in the signal-to-noise ratio, not the threshold.

## Results

Before presenting the results of our analysis modeling response times, we briefly describe the behavioral task as well as the previous findings relating to choice in this task.

### The Horizon Task

**Figure 2:**
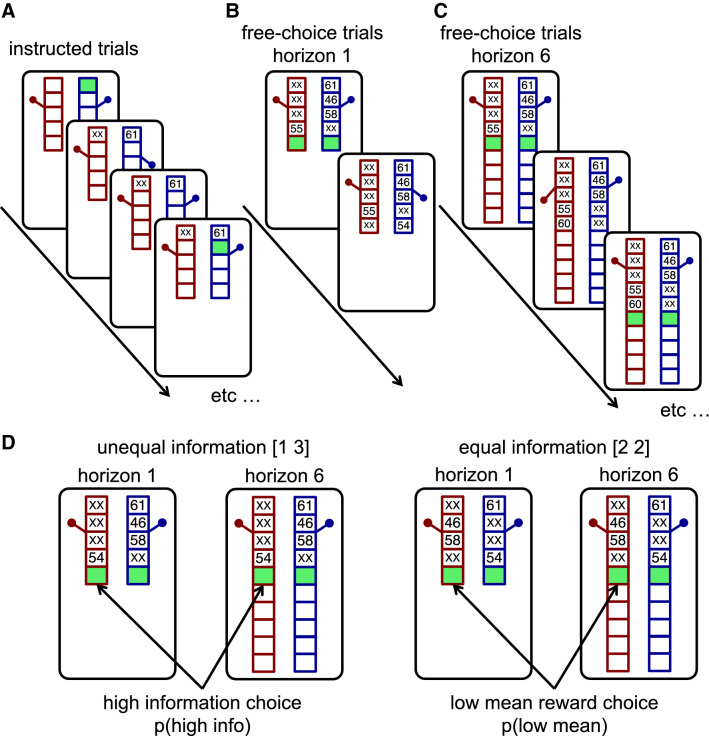
The Horizon Task. (**A**) Each game begins with four instructed trials in which participants are forced to pick the bandit with the green square. Note that the instructed trials are identical across horizon conditions except for the length of the bandits. (**B**,**C**) After the instructed trials, participants make free choices to the end of the game. In the short horizon condition, the game ends after one free choice (**B**). In the long horizon condition, participants make six free choices to complete the game (**C**). (**D**) The focus of analysis is the first free choice trial. On this trial, behavior is compared between two uncertainty conditions (unequal and equal) and two horizon conditions (short and long).

In the Horizon Task^4^ participants play a series of games, lasting 5 or 10 trials each corresponding to free response horizons of 1 and 6 (Fig. 2). During these games they make choices between two slot machines (also known as ‘one-armed bandits’). When chosen, a slot machine pays out a probabilistic reward sampled from a Gaussian distribution that is truncated and rounded so that the rewards are integers between 1 and 100 points. The means of the Gaussians are different for each option, such that one option is always better on average, but the standard deviation is the same for both options (8 points). The participants’ goal is to maximize their reward by exploiting the best option, but they are not told the means of the Gaussians, and thus they must explore both options to find out which one is best.

Critically, the first four trials of each game are ‘instructed’ trials (Fig. 2A). On these trials participants are told which option to play and forbidden from choosing the other option. This instruction allows us to control the information participants have before they make a free choice. In some games, participants are instructed to play one option once and the other three times. This sets up an ‘unequal’ information condition, in which participants are more uncertain about the option played once. In these ‘[1 3]’ games choosing this more uncertain option is more informative and we refer to this as the ‘high information’ choice. In other games, participants play both options twice to set up an equal (or [2 2]) information condition, in which both options are equally informative to play.

After the four instructed trials, participants make either 1 (short horizon condition) or 6 (long horizon condition) free choices (Fig. 2B,C). This horizon manipulation allows us to change the relative value of exploration and exploitation. When the horizon is short, participants should favor exploitation, because there is no time in the future to use any information gained by exploration. Conversely, in the long horizon condition, participants should favor exploration, at least at first. Thus, by contrasting behavior between horizon conditions *on the first free choice* of the game, the Horizon Task allows us to quantify directed and random exploration as changes in information seeking and behavioral variability with horizon (Fig. 2D).

### Information seeking and behavioral variability increase with horizon

**Figure 3:**
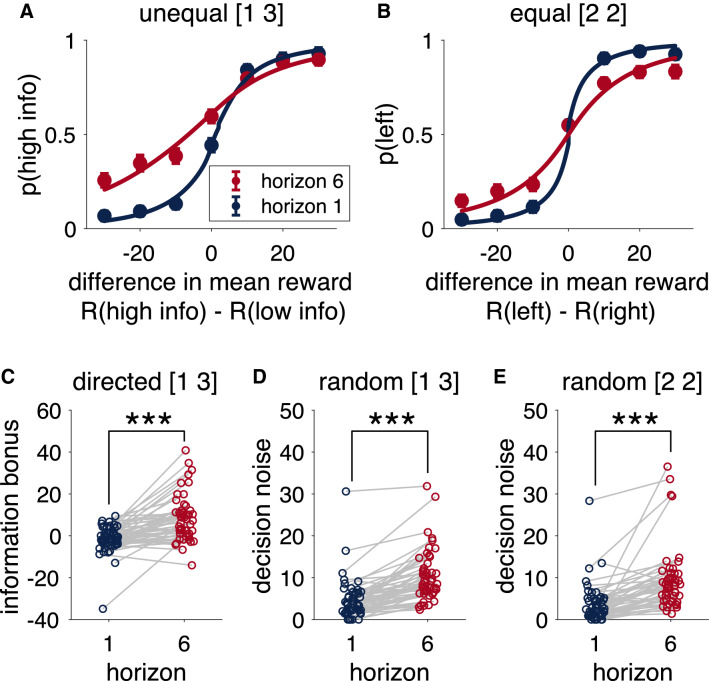
Choice behavior in the Horizon Task. (**A**,**B**) Choice curves showing the choice probability as a function of the difference in observed mean reward. (**A**) The probability of choosing the more informative option (i.e. the option played once during the instructed trials) as a function of the difference in observed reward between the more informative, $R(\text{ high } \text{ info})$ and less informative $R(\text{ low } \text{ info})$ bandits. (**B**) The probability of choosing the left bandit as a function of the difference in observed mean reward between the left and right options. (**C**,**D**,**E**) Fit parameter values, in units of points, from the logistic model showing the information bonus (**C**), as well as the standard deviation of the decision in the unequal (**D**) and equal (**E**) conditions. *** denotes a significant difference at $p<0.001$.

Choice behavior on the first free-choice trial of the Horizon Task shows clear evidence of directed and random exploration. Consistent with directed exploration, in the [1 3] condition participants are more likely to choose the more informative option in horizon 6 than horizon 1, as indicated by a shift in the indifference point of the choice curves (Fig. 3A). Consistent with random exploration, people’s behavior is less predictable (more random) in horizon 6 than horizon 1, as indicated by a lower slope of the choice curves in the horizon 6 condition (Fig. 3B).

In^4^ we quantified the choice curves using a simple logistic model. In this model, we write the choice probabilities in terms of the difference in observed mean reward of the two options, $\Delta R = R_{left} - R_{right}$, and the difference in information between the two options, $\Delta I = I_{left} - I_{right}$ (with information defined such that $\Delta I = +1$ when the left choice is more informative in the [1 3] condition, $\Delta I = -1$ when the right choice is more informative in the [1 3] condition, and $\Delta I = 0$ when neither option is more informative in the [2 2] condition). That is, we write the probability of choosing the left option as

1 $$\begin{aligned} p(\text{ choose } \text{ left}) = \frac{1}{1 + \exp \left( - \frac{\Delta R + A\Delta I + B}{\sqrt{2} \sigma } \right) } \end{aligned}$$

where the free parameters are: the information bonus, *A*, the spatial bias in favor of choosing left, *B*, and the standard deviation of the decision noise, $\sigma$. The spatial bias and noise parameters, *B* and $\sigma$, are fit separately in each of the information and horizon conditions. The information bonus *A* is fit in the two horizon conditions of the unequal information condition, which is the only condition in which it applies (there is no directed exploration when both options are equally uncertain).

Fit values of the information bonus and decision noise for each participant are plotted in Fig. 3C–E. As the horizon increases from 1 to 6, people exhibit both a larger information bonus, consistent with directed exploration (i.e. *A* increases, $t(45) = 6.53, p < 0.001$), and more decision noise, consistent with random exploration (i.e. $\sigma$ increases $t(45) = 7.66, p < 0.001$ for [1 3] condition; $t(45) = 5.78, p < 0.001$ for [2 2] condition). Thus we have previously concluded that humans use both directed and random exploration in the Horizon Task^4^.

### Response times suggest the decision is made on the first free-choice trial

**Figure 4:**
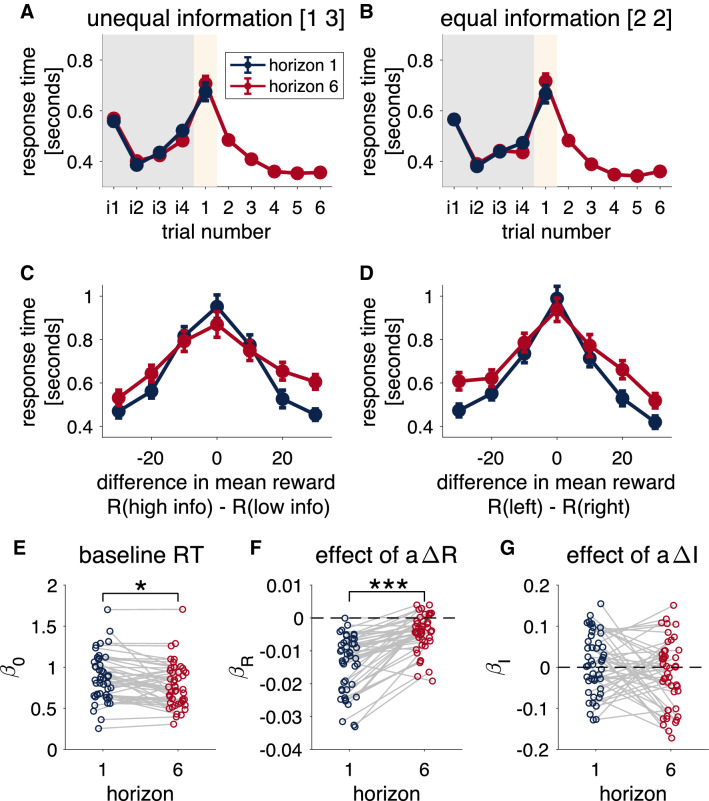
Response times in the Horizon Task. (**A**,**B**) Average response time as a function of trial number for the unequal (**A**) and equal (**B**) information conditions. Instructed trials are labeled i1-4, free-choice trials 1–6. (**C**,**D**) Response time as a function of difference in mean observed reward in the unequal (**C**) and equal (**D**) information conditions. (**E**,**F** **G**) Linear regression analysis showing the baseline response time (**E**), and the effects of $a{\Delta R}$ (**F**), and $a{\Delta I}$ (**G**) on response times.

Response times in the Horizon Task vary considerably with trial number (Fig. 4A,B). In the instructed trials, participants respond slowly on the first trial of each game (RT $\approx$ 0.6s), before speeding up for the remaining instructed trials (RT $\approx$ 0.4s). They then slow down on the first free-choice trial (RT $\approx$ 0.7s), before speeding up again on later trials. This pattern of response times suggests that people may be playing through the instructed trials as quickly as possible (at least once they decide to initiate the game on the first trial), and saving their deliberation for the first free-choice trial.

In line with the idea that people are deciding on the first free-choice trial, we find that response times on this trial are modulated by the difference in observed mean reward between the two options. In particular, people respond more slowly when the difference in reward is closer to zero (Fig. 4C,D). Such a pattern of response times is similar to that observed in value-based drift diffusion models of behavior^16,17^. In addition, the modulation of response time by reward seems to change between the two horizon conditions, with a weaker dependence of response time on $\Delta R$ in horizon 6 than horizon 1.

To quantify these effects we fit a linear regression model to the response times. In this model we assume that on each trial the response time is given by

2 $$\begin{aligned} RT = \beta _0 + \beta _R a \Delta R + \beta _I a \Delta I \end{aligned}$$

where *a* corresponds to the choice, or action, on the trial, coded as $+1$ for left and $-1$ for right. The regression coefficients $\beta _0$, $\beta _R$, and $\beta _I$ capture the baseline response time, the effect of reward on response time, and the effect of information on response time respectively.

Results from this regression model are shown in Fig. 4E–G. Both the baseline response time, $\beta _0$ (panel E), and the modulation of response time by reward, $\beta _R$ (panel F), change with horizon. In particular, people’s baseline response time becomes faster in the horizon 6 condition, while the dependence on reward gets weaker (i.e. less negative).

Taken together, this pattern of response times suggests that (1) people make their decision on the first free-choice trial, (2) modulate their response times according to reward, and (3) modulate their response times according to horizon. In the following sections we show how a value-based drift-diffusion model can account for these effects and shed light on the mechanisms underlying random exploration.

### Drift-diffusion model of the first free-choice trial

We model choice and response time on the first free choice of the Horizon Task using a value-based drift-diffusion model (e.g.^16–18^). In this model, we assume that the drift rate, bias, and threshold can all vary with the difference in reward ${\Delta R}$ and difference in information ${\Delta I}$. Thus we write

3 $$\begin{aligned} \mu& = c_0^\mu + c_R ^\mu {\Delta R}+ c_I^\mu {\Delta I} \end{aligned}$$

4 $$\begin{aligned} \beta& = c_0^\beta + c_R ^\beta {\Delta R}+ c_I^\beta {\Delta I} \end{aligned}$$

5 $$\begin{aligned} \alpha& = 2 L (c_0^\alpha + c_R ^\alpha {\Delta R}+ c_I^\alpha {\Delta I})-1 \end{aligned}$$

where the bias $\alpha$ relates to the starting point, as $x_0 = \alpha \beta$, and we use a logistic link function

6 $$\begin{aligned} L(x) = \frac{1}{1+\exp (-x)} \end{aligned}$$

to ensure that $\alpha \in [-1,1]$.

The free parameters of this model are the 9 coefficients, $c^i_j$ $(i \in \left\{ \mu , \beta , \alpha \right\} ; j \in \left\{ 0, R, I \right\} )$, and an additional non-decision time, $T_0$, in which the integration process does not occur. We further assume that each of these 10 parameters can change with horizon giving us 20 free parameters in total, per subject, as summarized in Table 1.

**Table 1:** Free parameters in the drift diffusion model.

Variable	Description
$c_0^\mu$	Baseline value of drift
$c_R^\mu$	Effect of ${\Delta R}$ on drift
$c_I^\mu$	Effect of ${\Delta I}$ on drift
$c_0^\beta$	Baseline value of threshold
$c_R^\beta$	Effect of ${\Delta R}$ on threshold
$c_I^\beta$	Effect of ${\Delta I}$ on threshold
$c_0^\alpha$	Baseline value of bias
$c_R^\alpha$	Effect of ${\Delta R}$ on bias
$c_I^\alpha$	Effect of ${\Delta I}$ on bias
$T_0$	Non-decision time

Note that all parameters are horizon dependent giving a total of 20 free parameters overall, per subject.

Note that there are two limitations to this model that arise from the linear dependence of the threshold on ${\Delta R}$ and ${\Delta I}$ in Eq. 4. First is the mathematical concern that the threshold, $\beta$, could become negative for certain values of ${\Delta R}$ or ${\Delta I}$, a situation for which the drift diffusion model’s behavior is undefined. Second is the psychological concern that the effects of ${\Delta R}$ and ${\Delta I}$ are asymmetric. That is, simply switching the locations of left and right bandits in Fig. 2D, without otherwise changing the decision, would lead to different thresholds (and different behavior) because the signs of ${\Delta R}$ and ${\Delta I}$ have changed. In the Supplementary Material, we fit a model in which threshold can vary as a function of the absolute value of ${\Delta R}$ and ${\Delta I}$. As shown there, repeating our analysis with this modified model does not change our scientific conclusions. In the main text, however, we persist with the linear dependence on ${\Delta R}$ and ${\Delta I}$ because of the stronger mathematical connection to the logistic choice model and the insights this connection provides.

### The logistic choice model is a special case of the drift-diffusion model

The form of the model described in Eqs. 3, 4, and 5can be mapped exactly to the logistic choice model (Eq. 1) in two special cases. While the full model is more general than either of these special cases, working through this mapping helps shed light on how random exploration could be controlled in the drift-diffusion model and provides a hint at what the pattern of response times in Fig. 4 might mean.

To make the mapping to the logistic model, we make use of the standard expression for choice probability in drift-diffusion models^19^. This allows us to write the choice probability as

7 $$\begin{aligned} p(\text{ choose } \text{ left}) = \frac{1}{1 + \exp (2 \beta \mu )} - \frac{1 - \exp \left( -2\alpha \beta \mu \right) }{\exp (2 \beta \mu ) - \exp (-2 \beta \mu )} \end{aligned}$$

If we assume that the initial condition is zero, i.e. $\alpha = 0$, then the second term on the right hand side goes to zero and the choice probabilities are logistic in $\beta$ and $\mu$. If we further assume that *either* $c_R ^\beta =c_I ^\beta = 0$ *or* $c_R ^\mu =c_I ^\mu = 0$, then this logistic function maps exactly onto the logistic choice function in Eq. 1.

In the case where $c_R ^\beta =c_I ^\beta = 0$, the threshold does not depend on either reward or information. In this case the choice probability becomes

8 $$\begin{aligned} p(\text{ choose } \text{ left}) = \frac{1}{1 + \exp \left( 2 c_0^\beta \left( c_0^\mu + c_R ^\mu {\Delta R}+ c_I^\mu {\Delta I}\right) \right) } \end{aligned}$$

By comparing Eq. 8 with the logistic choice function (Eq. 1), we can make the identification that the noise parameter in the logistic choice function is given by

9 $$\begin{aligned} \sigma = \frac{1}{2\sqrt{2}c^\mu _R c^\beta _0} \end{aligned}$$

Thus, if threshold is independent of reward and information, random exploration can be controlled by adjusting either the baseline threshold $c^\beta _0$, or the extent to which drift is modulated by reward $c^\mu _R$. We refer to this latter quantity as the signal-to-noise ratio (SNR), because it reflects the extent to which the value signal modulates the drift rate.

In the case where $c_R ^\mu = c_I^\mu = 0$, the drift rate is independent of the reward and information. Setting the drift-rate to be independent of reward is certainly not standard practice for value-based drift-diffusion models, however we include this possibility for completeness and let the data rule it in or out. In this case, the equation for choice probabilities becomes

10 $$\begin{aligned} p(\text{ choose } \text{ left}) = \frac{1}{1 + \exp \left( 2 {c_0^\mu } \left( c_0^\beta + c_R ^\beta {\Delta R}+ c_I^\beta {\Delta I}\right) \right) } \end{aligned}$$

with the identification

11 $$\begin{aligned} \sigma = \frac{1}{2\sqrt{2}c^\mu _0 c^\beta _R} \end{aligned}$$

Note that Eq. 11 involves different parameters to Eq. 9, suggesting that, in this case, random exploration can be controlled by either the baseline drift rate, $c^\mu _0$ or the effect of reward on threshold, $c^\beta _R$.

**Figure 5:**
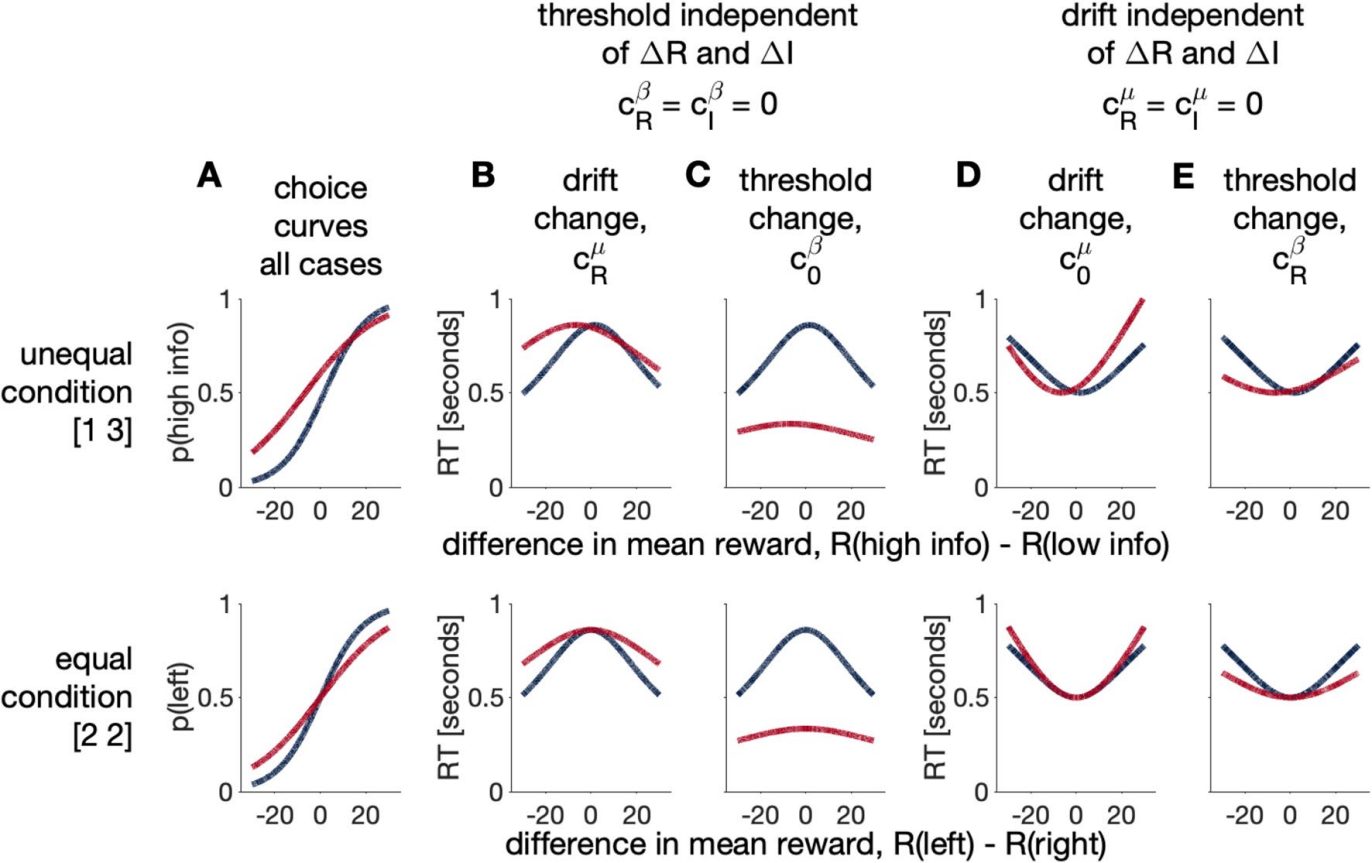
Qualitative predictions for the logistic versions of the drift-diffusion model using manually chosen parameters. Blue and red lines correspond to horizon 1 and 6, respectively. (**A**) With appropriately chosen parameter values, there are four ways in which the diffusion model reduces to the logistic choice model. All four models have identical choice behavior. However, they differ in the patterns of response times that they produce (**B**,**C**,**D**,**E**). (**B**,**C**) In the case where threshold is independent of reward and information, $c^\beta _R = c^\beta _I = 0$, there is a maximum response time. A change in drift with horizon leads to slower response times in horizon 6 than horizon 1 (**B**), while a change in threshold with horizon leads to faster response times in horizon 6 than horizon 1 (**C**). (**D**,**E**) When drift rate is independent of reward and information, $c^\mu _R = c^\mu _I = 0$, there is a minimum response time. A change in baseline drift with horizon leads to (mostly) an increase in response times in horizon 6, while a change in threshold leads to a decrease in response times in horizon 6. See section 3 of the Supplementary Material for parameter values.

Thus, at the level of choice, the drift-diffusion model suggests there are four different parameters by which random exploration could be controlled: (1) the baseline threshold, $c^\beta _0$, (2) the effect of ${\Delta R}$ on drift rate, $c^\mu _R$ or SNR, (3) the effect of ${\Delta R}$ on threshold, $c^\beta _R$, and (4) the baseline drift rate $c^\mu _0$. However, because all four of these mechanisms lead to identical logistic choice curves (Fig. 5A), it is impossible to distinguish between these mechanisms using choice data alone. For that we need response times.

### Different mechanisms for random exploration lead to different patterns of response times

While the different mechanisms for random exploration lead to identical choices, they lead to markedly different patterns of response times. In particular, using the standard equation for the response times in drift-diffusion models^19^ we can write the response time as

12 $$\begin{aligned} RT = T_0 + \frac{\beta }{\mu } \tanh (\beta \mu ) + \frac{\beta }{\mu } \frac{2 \left( 1 - \exp \left( -2\alpha \beta \mu \right) \right) }{\exp (2 \beta \mu ) - \exp (-2 \beta \mu )} - \alpha \beta \mu \end{aligned}$$

When $\alpha = 0$, this reduces to

13 $$\begin{aligned} RT = T_0 + \frac{\beta }{\mu } \tanh (\beta \mu ) \end{aligned}$$

In the case where threshold does not depend on reward or information, i.e. $c_R^\beta =c_I^\beta =0$, this expression for response times becomes

14 $$\begin{aligned} RT = T_0 + \left( \frac{c^\beta _0}{c^\mu _0 + c^\mu _R\Delta R + c^\mu _I \Delta I} \right) \tanh \left( c^\beta _0\left( c^\mu _0 + c^\mu _R\Delta R + c^\mu _I \Delta I\right) \right) \end{aligned}$$

Conversely, when drift does not depend on reward or information, i.e. $c^\mu _R = c^\mu _I = 0$, the response times are given by

15 $$\begin{aligned} RT = T_0 + \left( \frac{c^\beta _0 + c^\beta _R \Delta R + c^\beta _I \Delta I}{c^\mu _0} \right) \tanh \left( {c^\mu _0}\left( c^\beta _0 + c^\beta _R \Delta R + c^\beta _I \Delta I \right) \right) \end{aligned}$$

Note the different dependence on ${\Delta R}$ in Eqs. 14 and 15. In Eq. 14, *RT* has a global maximum at

16 $$\begin{aligned} {\Delta R}^* = -\frac{c^\mu _I {\Delta I}+ c^\mu _0}{{c^\mu _R}} \end{aligned}$$,

and decreases as ${\Delta R}$ moves away from this point (Fig. 5B,C). In Eq. 15, *RT* has a global minimum at

17 $$\begin{aligned} {\Delta R}^* = -\frac{c^\beta _I {\Delta I}+ c^\beta _0}{{c^\beta _R}} \end{aligned}$$,

and increases as ${\Delta R}$ moves away from this point (Fig. 5D,E). Comparison with human behavior in Fig. 4 already suggests that the models where $c^\mu _R = c^\mu _I = 0$ are not a good description of behavior.

How the response times change with horizon allows us to further distinguish between the two remaining models when $c^\beta _R = c^\beta _I = 0$. If random exploration is controlled by the effect of ${\Delta R}$ on drift, $c^\mu _R$, then response times in horizon 6 will be slower than horizon 1 because behavioral variability is increased by reducing $c^\mu _R$ in horizon 6 (Fig. 5B). Conversely, if random exploration is controlled by the baseline threshold, $c^\beta _0$, then response times in horizon 6 will be faster than horizon 1 because behavioral variability is increased by reducing $c^\beta _0$ (Fig. 5C).

Comparison with human behavior in Fig. 4 suggests that response times are slower in horizon 6 (except for ${\Delta R}= 0$ in the unequal condition), suggesting a drift mechanism for random exploration in which $c^\beta _R$, the signal-to-noise ratio, is reduced in horizon 6. Of course, this qualitative analysis of response times can only take us so far, to be sure that behavioral variability is controlled by SNR we need to explicitly fit the full model.

### Model fitting suggests that both signal-to-noise ratio and threshold change with horizon

While the qualitative analysis of response times presented above suggests a drift mechanism for random exploration controlled by the signal-to-noise ratio, $c^\mu _R$, it does not rule out the possibility that the other parameters also play a role in random exploration. To test this possibility, and to relax the assumption that the starting point is always zero, we fit the full 20-parameter model to the behavior.

For each subject in each horizon condition, we fit parameters using the a maximum likelihood approach (see Methods). In the Supplement we show that this method leads to excellent parameter recovery in this task (Supplementary Figure S2) and that the parameters estimated with this approach almost exactly match those computed using the Bayesian HDDM python toolbox^20^ (Supplementary Figure S1).

**Figure 6:**
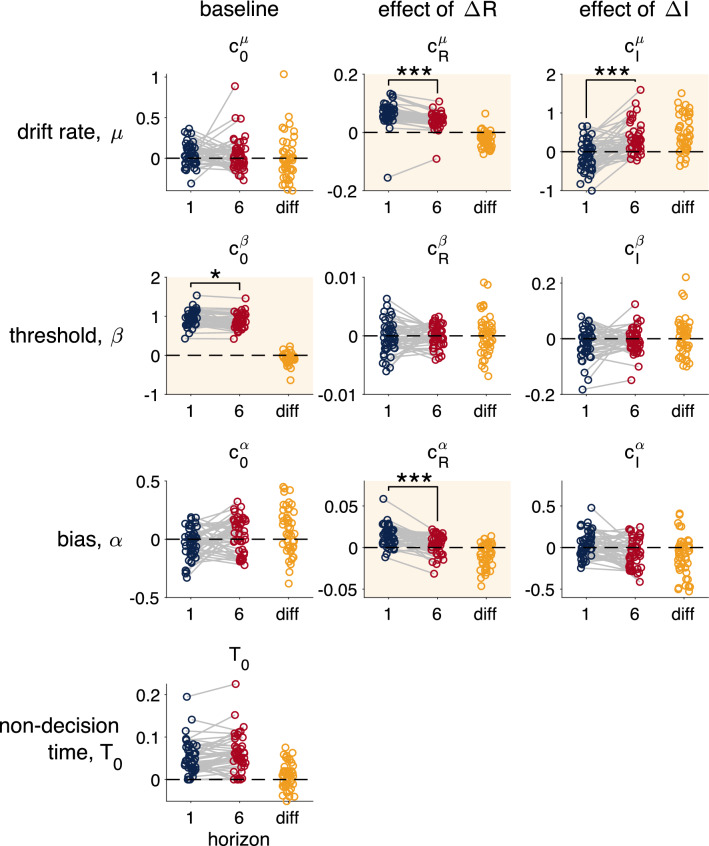
Fit drift-diffusion model parameters in the Horizon Task. Each row corresponds to a different parameter in the drift-diffusion model (drift rate, threshold, bias and non-decision time). Each column corresponds to a different component of each parameter (its baseline value, how it changes with reward, and how it changes with information). Parameters that change significantly between horizon 1 and horizon 6 are highlighted with a yellow background. * denotes a significant horizon difference at $p<0.01$, *** at $p<0.001$. All significance checks were Bonferroni corrected for 10 multiple comparisons.

In Fig. 6 we plot the fit parameter values from the maximum likelihood procedure for each subject in the two horizon conditions, as well as the difference in parameter value (e.g. $T_0(\text{ horizon } \text{6 }) - T_0(\text{ horizon } \text{1 })$). Consistent with directed exploration, adding an information bonus to the drift rate, $c^\mu _I$ increases with horizon ($t(45) = 6.55$, $p < 0.001$). That is, participants show greater drift towards the more informative option in horizon 6 than they do in horizon 1.

Consistent with random exploration making behavior more variable in horizon 6, both $c^\mu _R$ ($t(45) = 6.65$, $p < 0.001$) and $c^\beta _0$ decrease with horizon ($t(45) = 3.55$, $p < 0.001$). Unlike the qualitative analysis, this suggests that *both* drift changes, $c^\mu _R$, and threshold changes, $c^\beta _0$, may underlie random exploration.

In addition to these effects on drift rate and threshold, we also see an effect of horizon on the bias. In particular, the effect of reward on bias, $c^\alpha _R$ is reduced in horizon 6 relative to horizon 1. This could reflect processing of reward before the first free-choice trial, which is entirely possible given that participants gain information throughout the instructed trials.

Beyond the parameters that change with horizon, there were several parameters that were not significantly different from zero in either horizon condition: the baseline drift rate, $c_0^\mu$, the baseline starting point, $c^\alpha _0$, the effect of ${\Delta R}$ on threshold, $c_R^\beta$, the effect of ${\Delta I}$ on threshold, $c_I^\beta$, and the effect of ${\Delta I}$ on bias, $c^\alpha _I$.

**Figure 7:**
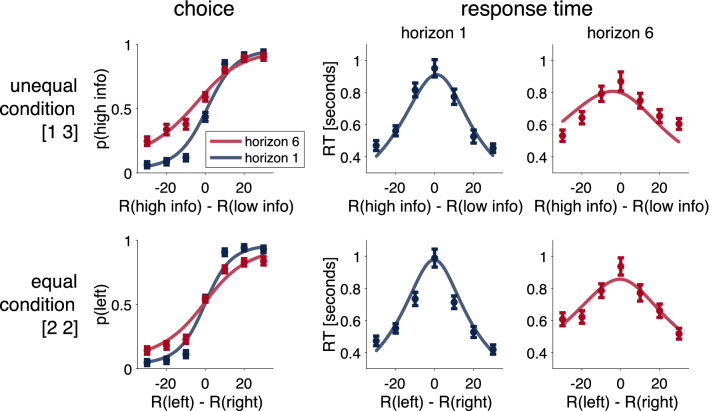
The drift-diffusion model (solid lines) captures the main qualitative effects of human choice and response time data (dots) in all conditions of the experiment.

Finally, comparing the theoretical choice and response time curves (Eqs. 7 and 12using the fit parameter values) shows that the drift-diffusion model captures the main qualitative features of the choice and response time curves (Fig. 7).

### Sensitivity analysis suggests that random exploration is dominated by changes in SNR

The results of model fitting suggest that both signal-to-noise ratio, $c^\mu _R$, and threshold, $c^\beta _0$, change with horizon and thus may be behind the changes in behavioral variability associated with random exploration. However, a horizon-based change in parameter value does not, in and of itself, indicate how large the effect on behavioral variability will be. Thus we next examine the extent to which each process (SNR change or threshold change) contributes to the change in variability with horizon.

To do this we make use of the relationship between the drift-diffusion model and the logistic choice model. In particular, we note that in the diffusion model fits (Fig. 6), the baseline bias, $c^\alpha _0$ is approximately zero, as are $c^\beta _R$, $c^\beta _I$. This suggests that for the fit parameter values, the drift-diffusion model approximates the logistic model with noise given by Eq. 9; i.e.

18 $$\begin{aligned} \sigma = \frac{1}{2\sqrt{2}c^\mu _R c^\beta _0} \end{aligned}$$

To test this relationship between $\sigma$, $c^\mu _R$, and $c^\beta _0$, we computed the predicted noise parameter from the fit drift-diffusion model parameters (RHS of Eq. 9) and compared it to the noise parameter $\sigma$ from the logistic model (LHS Eq. 9). These quantities were tightly coupled ($r > 0.76$, $p < 0.001$ for both horizon conditions, Supplementary Figure S3) implying that the approximations required to derive Eq. 9 hold.

**Figure 8:**
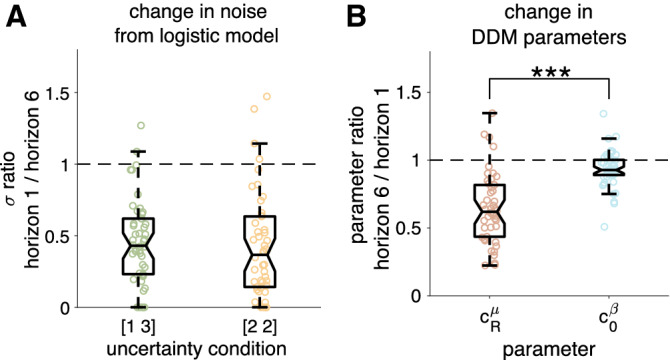
Sensitivity analysis. (**A**) The ratio of decision noise in horizon 6 compared to decision noise in horizon 1 computed using the $\sigma$ parameter from the logistic model for the equal and unequal conditions. In both cases the median ratio is just over 2. (**B**) The ratios of drift-diffusion parameters, $c^\mu _R$ and $c^\beta _0$, in horizon 1 to those in horizon 6. Note that for comparison with panel (**A**), the ratios are reversed (i.e. horizon 1:horizon 6 for (**B**) instead of horizon 6:horizon 1 for (**A**), see Eq. 18). *** indicates a significant difference at $p<0.001$.

If Eq. 9 does hold, then this implies that the relative change in random exploration between horizon 1 and 6 can be written as

19 $$\begin{aligned} \frac{\sigma (\text{ horizon } \text{1 })}{\sigma (\text{ horizon } \text{6 })} = \frac{c^\mu _R(\text{ horizon } \text{6 })c^\beta _0(\text{ horizon } \text{6 })}{c^\mu _R(\text{ horizon } \text{1 })c^\beta _0(\text{ horizon } \text{1 })} \end{aligned}$$

and therefore that the relative contribution of $c^\mu _R$ and $c^\beta _0$ to random exploration is determined by the relative amount by which each parameter changes from horizon 1 to horizon 6. Computing these ratios for the fit parameters, we find that $c^\mu _R$ changes by a larger amount ($c^\mu _R(\text{ horizon } \text{6 }) / c^\mu _R(\text{ horizon } \text{1 })\sim 0.645$) than $c^\beta _0$ ($c^\beta _0(\text{ horizon } \text{6 }) / c^\beta _0(\text{ horizon } \text{1 }) \sim 0.933$, Fig. 8). This is to be compared with a change in logistic decision noise of around 0.4 ($\sigma (\text{ horizon } \text{1 }) / \sigma (\text{ horizon } \text{6 }) = 0.456$ in the [1 3] condition and 0.426 in the [2 2] condition). This suggests that random exploration is primarily driven by the change in signal-to-noise ratio, $c^\mu _R$, and not the change in baseline threshold.

## Discussion

In this paper we developed a drift-diffusion model of explore–exploit decision making to investigate the mechanisms underlying random exploration. This model includes the logistic model of exploration used in previous work as a special case, and suggests four different mechanisms by which the control of behavioral variability for random exploration could be achieved. While it is impossible to distinguish between these mechanisms using choice data alone, they lead to qualitatively different patterns of response times. Thus, by fitting the model to response time data in a popular explore–exploit task, we found evidence that random exploration was driven by two of these mechanisms: changes in drift rate, specifically how strongly reward modulated drift, the signal-to-noise ratio $c^\mu _R$, and baseline threshold, $c^\beta _0$. Further analysis suggested that the change in drift dominates, accounting for most of the change in variability associated with random exploration.

Taken together, our findings suggest that random exploration is primarily driven by a change in the signal-to-noise ratio with which reward information is encoded in the brain. That is, when it valuable to explore, the representation of reward cues—or at least the extent to which these cues are incorporated into the decision—is reduced, leading to weaker drift rates, slower response times, and more random exploration overall. Such a mechanism is consistent with recent reports that neural variability is increased when monkeys are in an exploratory ‘state’^7^ and that fMRI signal variability is increased in motor cortex when participants randomly explore^21^. This SNR mechanism is also consistent with older findings from the bird song literature, in which increased variability in song during practice, is associated with increased neural variability^22–25^. More generally, such a signal-to-noise ratio mechanism also gives a point of contact with other theories of how environmental noise supports information processing in the brain and other physical systems^26–30^.

A natural next step will be to leverage the model reductions suggested by our results. Indeed, our analysis fits 20 parameters per subject (10 per horizon condition), and in order to obtain robust fits we needed at least 130 games per horizon condition per subject (which limited our final sample size). Moving forward, however, we can make assumptions that decrease this total number of parameters per subject (e.g. assume that $c_I^\beta$ is the same for both horizon conditions), which opens the door for fitting participants with fewer trails. In addition to replicating our findings, such improvements would open the door to future studies in populations limited to low numbers of trials (e.g. aging or mentally disordered subjects).

Future investigation may also seek an explanation for the behavior on the rest of the Horizon Task, as opposed to the first free response trial analyzed here. In other words, how are the various parameters of the DDM “learned” in the Horizon task? This would require modeling both the learning and decision processes at work. Ongoing research in this area aims to link reinforcement learning models with sequential sampling models that are mathematically equivalent to the DDM used in this study (see^31,32^ for recent reviews). Assuming that human agents exert control over their DDM parameters, future work could take a similar reinforcement learning and DDM approach to understand the dynamics that give rise to the signal-to-noise mechanism revealed in the current study.

Another key question is whether the change in signal-to-noise ratio is driven by a change in the signal, a change in the noise, or a change in both. Because of a scaling condition in the equations of the drift-diffusion model, signal and noise are confounded in behavior, and this question is impossible to address with our data. However, with neuroimaging and electrophysiology it should be possible to separately measure signal and noise in the brain and resolve this question. If increased variability is caused by reduced signal, then the strength of reward signals in the brain should be reduced when exploration is valuable. Conversely, if increased variability is due to increased noise, then the average reward signal will be the same, but the variability in the signal (from trial to trial and within a single trial) will be larger.

Lastly, we ask what exactly is the ‘evidence’ that is being integrated over time in the explore–exploit choice? While evidence has a relatively clear meaning for perceptual decisions in classic drift-diffusion models^19^, it is less clear for their value-based cousins. One intriguing possibility for explore–exploit choices, is that the evidence that is being integrated corresponds to mental simulations of possible futures. Indeed, we have recently proposed such a mental simulation model of explore–exploit choices in a different task^33^. In this ‘Deep Exploration’ model of explore–exploit behavior, decisions are made by mental simulation of plausible future outcomes (e.g. what outcome might I receive if I explore first, what would I do then, etc ...). Each simulation generates a sample from the expected value of exploring or exploiting, and the decision is made by accumulating these samples to pick the option with the highest average simulated value. In our previous work, we considered the case where the number of simulations was fixed, but the model, at least in principle, is readily extended to the case where the decision is made by a threshold crossing process instead. A major goal for future work will therefore be to explicitly connect the drift-diffusion model presented here with the Deep Exploration account in^33^ to create a complete theory of the dynamics of explore–exploit choice.

## Methods

### Participants

Data used in this paper come from two previous published data sets: 30 participants (11 male, 20 female, ages 18–24, mean 19.7) from the original Horizon Task paper^4^ and an additional 30 participants (9 male, 20 female, ages 18–50, mean 22.7) who made up additional young adults in^34^. Both data sets were acquired at Princeton University. In both cases participants gave informed consent and the studies were approved by the Princeton Institutional Review Board. All experiments were performed in accordance with relevant guidelines and regulations.

### Exclusion criteria

In order to obtain meaningful parameters from the drift-diffusion model, we excluded trials in which participants responded to quickly (response time less than 0.1 seconds) or too slowly (response time less than 3 seconds). After this exclusion of trials, we then excluded participants who had less than 131 remaining trials for either horizon condition. This left 46 participants (10 male, 36 female, ages 18–28, mean 20.7) for the main analysis.

### The horizon task

In the Horizon Task (Fig. 2), participants choose between two slot machines, or one-armed bandits. When chosen, the slot machines pay out rewards, sampled from Gaussian distributions that are truncated (to lie between 1 and 100 points) and rounded (to be integers). The means of the Gaussians are different for each machine such that one machine is always better on average. In particular, the mean of one machine, randomly assigned to be on the left or right, is always set to either 40 or 60 points, while the mean of the other machine is set to be one of 4, 8, 12, 20, or 30 points higher or lower. The standard deviation of the Gaussians is the same for both options and is set to 8 points. In the instructions, participants are told that one option is always better and that the variability of the bandits (i.e. the standard deviation) remains the same over the entire experiment.

Trials in the Horizon Task are lumped together into ‘games’ lasting either 5 or 10 trials each. For each game the means of the Gaussians are selected using the process described above, but then remain constant for the remainder of the game before changing again for the next game. The duration of each game is indicated by the length of the bandits, which contain ‘slots’ that record the outcome of each trial. Short bandits, with 5 slots for the outcomes indicate short games. Long bandits, with 10 slots for the outcomes indicate long games.

Each game begins with four instructed trials, during these trials participants are instructed to play one option and are unable to play the other. By controlling which options participants play on these trials, we use the instructed trials to setup one of two information conditions: an unequal condition, in which one option is played once and the other three times (also known as the [1 3] condition), and an equal condition, in which both options are played twice (aka the [2 2] condition).

After the instructed trials, depending on the length of the game, participants have either 1 (5-trial games, short horizon condition) or 6 (10-trial games, long horizon condition) free choices between the two bandits. This horizon manipulation is the critical component of the Horizon Task. When the horizon is long, exploration is valuable, but when the horizon is short participants should always exploit. Thus by contrasting behavior between horizon conditions on the first free-choice trial of each game, we can quantify the directed and random exploration as the change in information seeking and behavioral variability with horizon.

### Logistic model of choice

In the logistic model of choice, we assume that choices are generated according to

20 $$\begin{aligned} p(\text{ choose } \text{ left}) = \frac{1}{1 + \exp \left( - \frac{{\Delta R}+ A{\Delta I}+ B}{\sqrt{2} \sigma } \right) } \end{aligned}$$

where ${\Delta R}= R_{left} - R_{right}$ is the difference in the mean of the observed rewards for the left and right options, and ${\Delta I}$ is the difference in information. ${\Delta I}$ is defined categorically such that ${\Delta I}= +1$ when left is the more informative option in the [1 3] condition, i.e. when left has been played once during the instructed trials, ${\Delta I}= -1$ when right is the more informative option in the [1 3] condition, and ${\Delta I}= 0$ in the [2 2] condition.

The parameters of the logistic model are: the information bonus, *A*, the side bias, *B*, and the standard deviation of the decision noise $\sigma$. These parameters are fit to separately to the choices on the first free-choice trial for each horizon condition (*A*) and each horizon $\times$ uncertainty condition (*B* and $\sigma$). Together this gives 6 free parameters.

### Fitting the logistic model

Following^4^, we fit the logistic model using a maximum a posteriori approach. In particular, to avoid excessively large parameter values, we used the following exponential prior on $\sigma$ (with length scale 20), and a Gaussian prior on *A*, with mean 0 and standard deviation 20. Maximization was performed using the fmincon function in Matlab.

### Linear model of response times

In the linear model of response times we assume that the response is given by

21 $$\begin{aligned} RT = \beta _0 + \beta _R a \Delta R + \beta _I a \Delta I \end{aligned}$$

where *a* corresponds to the choice, or action, on the trial, coded as $+1$ for left and $-1$ for right. The regression coefficients $\beta _0$, $\beta _R$, and $\beta _I$ capture the baseline response time, the effect of reward on response time, and the effect of information on response time respectively. This model was fit to participant data using the glmfit function in Matlab.

### Drift diffusion model

We model the response times using a model based on the well-known drift-diffusion model (DDM, Fig. 1), originally introduced by^15^, which has been used to model a variety of 2AFC paradigms^19,35–37^. More recently, such models have been successfully employed in studying value based decisions more similar to those in the present task e.g.^16–18^. Our model is an adaptation of what is commonly called the ‘simple’ or ‘pure’ DDM^16,19^.

At every instant, the model encodes a relative value signal ($X$) representing the accumulated ‘evidence’ favoring the hypothesis that the left bandit has a higher value ($X>0$) than the bandit on the right ($X<0$). This relative value signal evolves according to a simple stochastic differential equation, written in Itô form as:

22 $$\begin{aligned} dX(t) = \mu dt + cdW(t) \end{aligned}$$

where $\mu dt$ is a drift rate representing the average change in evidence supporting a left ($\mu >0$) or right ($\mu <0$) response and $cdW(t)$ is Gaussian distributed ‘white noise’ with mean 0 and variance $c^2 dt$.

A choice is made when the relative value crosses a threshold at $+\beta$ for left and $-\beta$ for right. We also include a fixed nondecision time $T_0$, an initial period of the response time when there is no accumulation happening (i.e. $X(t)$ does not change for $t \in [0,T_0]$).

Finally, the accumulation starts at some initial state of evidence: $X(0) = X_0$ which we usually write as $X_0 = \alpha \beta$ where $-1\le \alpha \le 1$. In this context, we call $\alpha$ the ‘bias’. It is commonly known that one of $\mu ,\beta ,c$ can be fixed without changing the model’s response time distributions^19^, we thus fix $c= 1$. Our formulation of the simple/pure DDM then has 4 parameters: $\mu , \beta , \alpha , T_0$. Our modeling effort, then, is to incorporate the elements of the Horizon Task into these key parameters.

To model behavior on the first free-choice of each game, we assume that the drift rate, threshold, and bias, can all vary with difference in reward ${\Delta R}$ and difference in information ${\Delta I}$. Thus we write

23 $$\begin{aligned} \mu& = c_0^\mu + c_R ^\mu {\Delta R}+ c_I^\mu {\Delta I} \\ \beta& = c_0^\beta + c_R ^\beta {\Delta R}+ c_I^\beta {\Delta I}\\ \alpha& = 2 L (c_0^\alpha + c_R ^\alpha {\Delta R}+ c_I^\alpha {\Delta I})-1 \end{aligned}$$

where *L* is a logistic link function (main text, Eq. 6). This yields 9 free parameters to describe the baseline value, effect of reward, and effect of information on each of drift, threshold and bias. Combined with the non-decision time, $T_0$, this gives us 10 free parameters that we fit each each horizon condition, giving 20 free parameters overall.

### Fitting the drift diffusion model

We fit the drift-diffusion model to participant choices and response times using a maximum likelihood approach. This approach centered on the method of^38^ for fast and accurate computation of the first passage time distribution of the drift-diffusion process. Fits were performed in Matlab using the fmincon function. All codes and data used to reproduce the figures and analysis are available at https://github.com/sffeng/horizon_ddm.

## Supplementary Information

Supplementary Information
