# Supplementary material for: The dynamics of explore–exploit decisions reveal a signal-to-noise mechanism for random exploration
Source: Sci Rep. 2021 Feb 4;11:3077. doi: 10.1038/s41598-021-82530-8 (PMC7862437; doi:10.1038/s41598-021-82530-8)
Supplement: Supplementary file 1 — Supplementary Information [file 41598_2021_82530_MOESM1_ESM.pdf]

Supplementary Material: The dynamics of explore-exploit decisions reveal a signal-to-noise mechanism for random exploration

Samuel F. Feng[1,4], Siyu Wang[2], Sylvia Zarnescu[2], and Robert C. Wilson[2,3,*]

[1]Department of Mathematics, Khalifa University of Science and Technology, Abu Dhabi, UAE
[2]Department of Psychology, University of Arizona, Tucson AZ USA
[3]Cognitive Science Program, University of Arizona, Tucson AZ USA
[4]Khalifa University Centre for Biotechnology, Khalifa University of Science and Technology, Abu Dhabi, UAE
[5]Correspondence to: bob@arizona.edu

1 Fitting the drift-diffusion model using the HDDM

In addition to fitting with the maximum likelihood approach, we also used the HDDM python toolbox [20], which is known to have strong performance in situations with relatively low numbers of trails (compared to the number of free parameters) [39]. Our fits used 200,000 MCMC samples (discarding 20,000 for burn-in), and typical heuristics were checked to further suggest that our Markov chains had converged (e.g. Markov chain error $< 1\%$ and visual inspection of the converged chains). Average parameters fit using the HDDM were almost identical to those found using the maximum likelihood approach (Figure S1). Thus, in the main text we focused on the simpler and much faster (30s for MLE vs 5 days for MCMC to fit on a laptop) maximum likelihood fits. All codes and data used to reproduce the figures and analysis are available at `https://github.com/sffeng/horizon_`

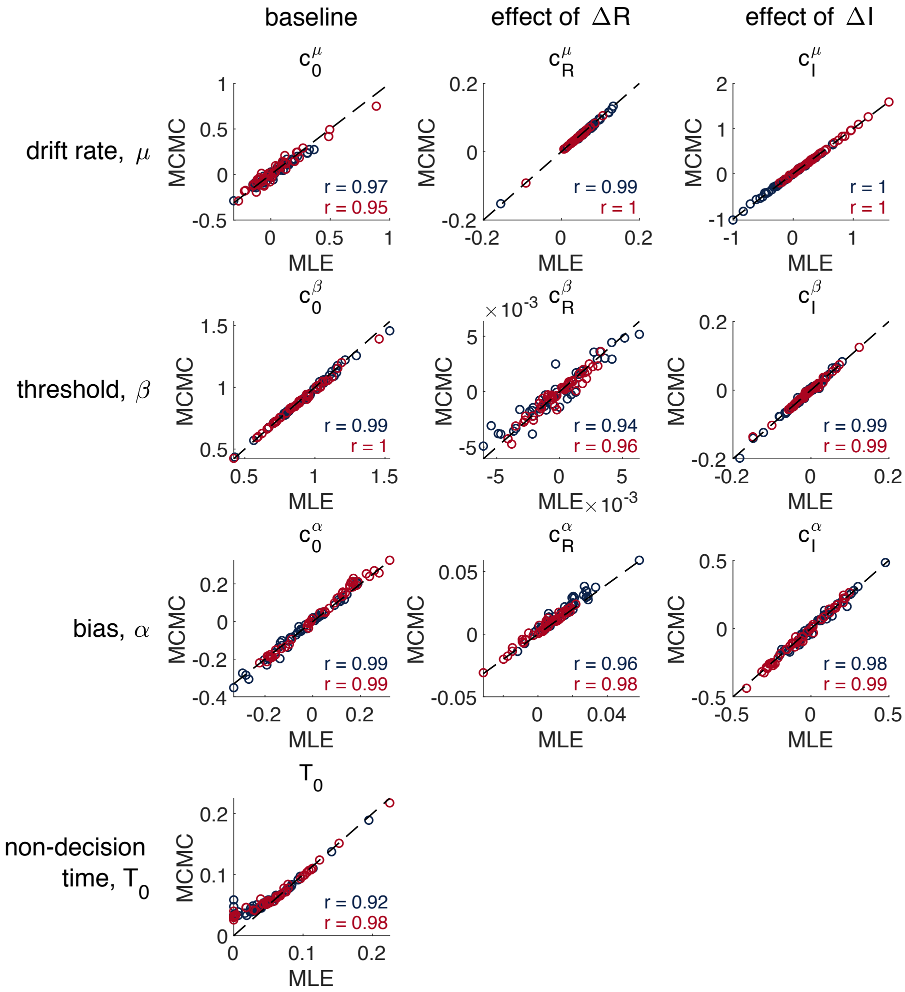

Figure S1: Comparison between MLE and MCMC parameter values for the full model from horizon 1 (blue) and horizon 6 (red) games.

2 Parameter recovery

Parameter recovery [40] was performed by fitting simulated data. In particular, we simulated 46 participants worth of data using the same parameters that we found by fitting real data. This simulated data was fit in exactly the same way as the original data set, using the maximum likelihood approach. We then compared the recovered parameters to the ground truth parameters from simulation. As shown in Figure S2, parameter recovery is excellent for this model in this task. In particular, recovery of the most important parameters, as far as random exploration is concerned (c_0^β and c_R^μ) is near perfect (correlation between simulated and fit parameters is greater than 0.93 in all cases).

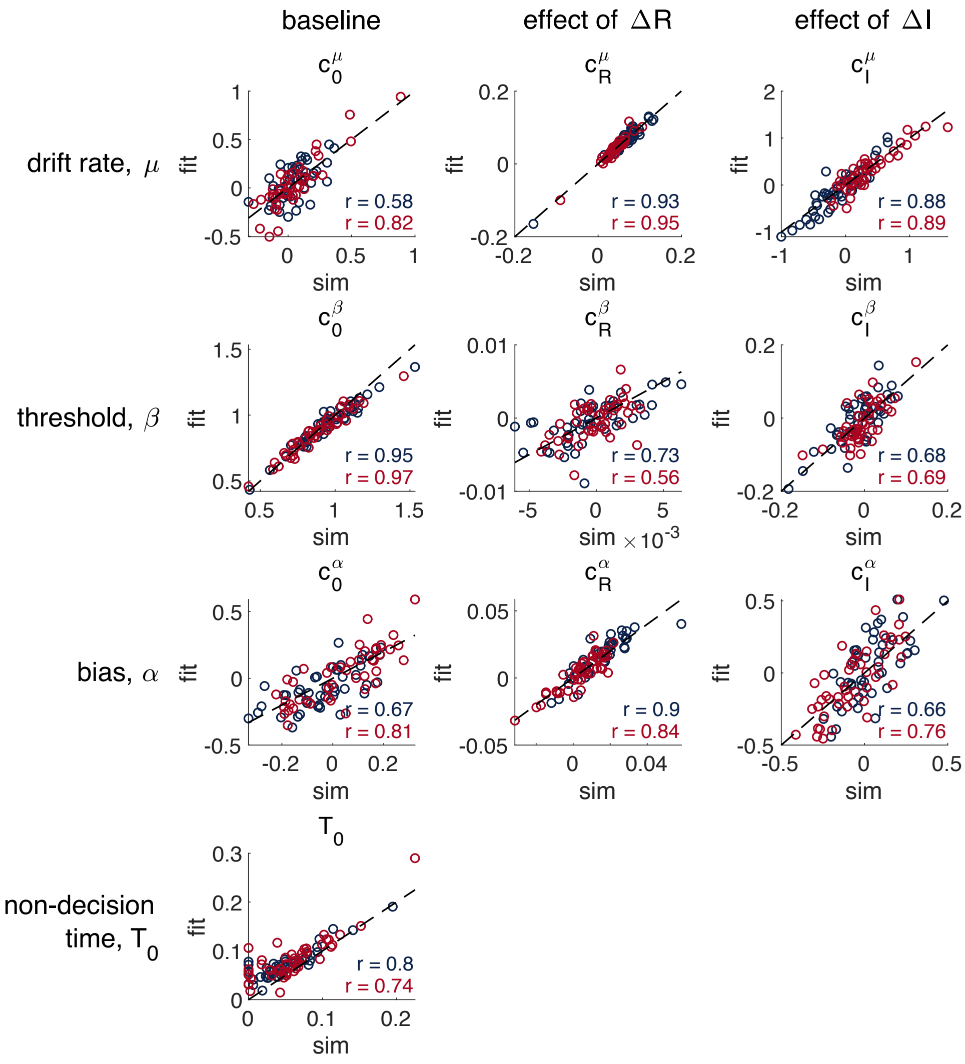

Figure S2: Parameter recovery for the full model with MLE fits for horizon 1 (blue) and horizon 6 (red) games.

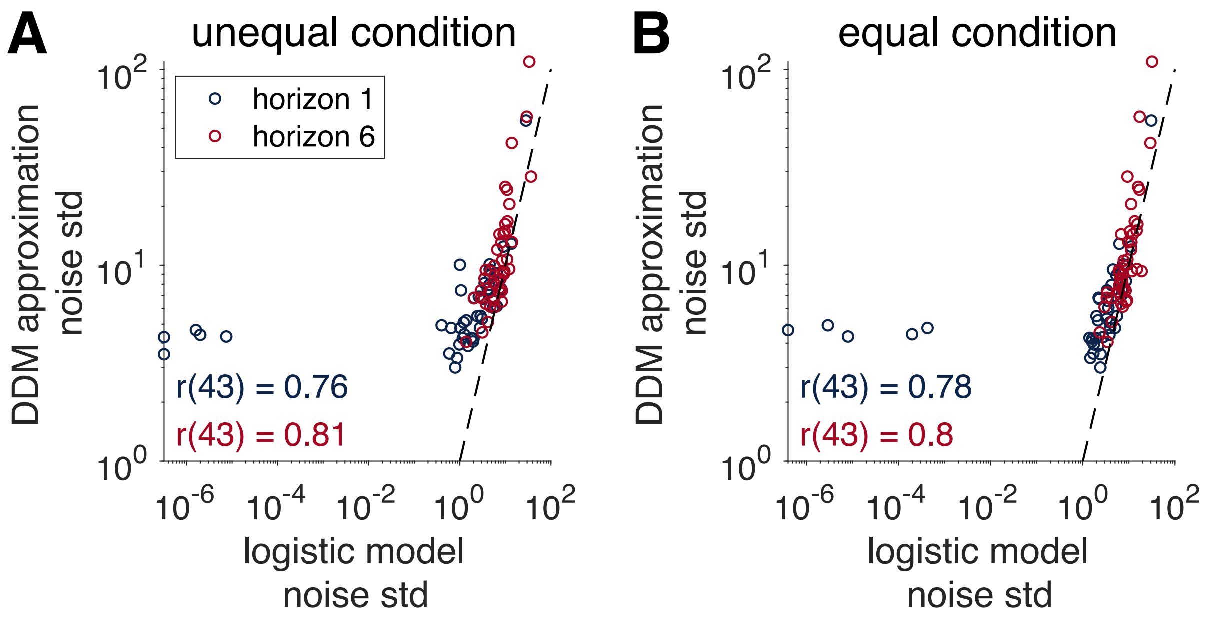

Figure S3: Comparison between noise estimated from the logistic model and approximate noise from Equation 9. Note one subject, who had negative drift rate parameters, was excluded from this analysis.

3 Parameter Values for Figure 5

The parameter values for Figure 5, columns B through E were chosen by hand to visually represent the different qualitative predictions of the logistic version of the drift-diffusion model. The bias parameters were fixed at 0: $c_0^\alpha = c_R^\alpha = c_I^\alpha = 0$ and non-decision time was fixed at $T_0 = 0.05$. The remaining parameters are given in Table S1.

4 Model with threshold dependent on absolute value of ΔR and ΔI

As mentioned in the main text, having the threshold be linearly dependent on ΔR and ΔI could be problematic both from a mathematical point of view (negative thresholds leading to undefined behavior) and psychological point of view (threshold depends on spatial location of bandits). To avoid these problems we fit a modified form of the model in which the threshold depends on the absolute value of ΔR and ΔI.

$$\beta = c_0^\beta + c_R^\beta |\Delta R| + c_I^\beta |\Delta I| \quad \text{(S1)}$$

First we fit the model to simulated data to check parameter recovery for this modified model. As shown in Figure S4, parameter recovery was similarly good for this model as

Table S1: Parameter values for Figure 5 columns B through E

	threshold independent, $c_R^\beta = c_I^\beta = 0$			
	B: drift change, c_R^μ		C: threshold change, c_0^β	
	Horizon 1	Horizon 6	Horizon 1	Horizon 6
c_0^μ	0	0	0	0
c_R^μ	0.06	0.0356	0.06	0.06
c_I^μ	-0.1	0.237	-0.1	0.4
c_0^β	0.9	0.9	0.9	0.5333
c_I^β	0	0	0	0
c_R^β	0	0	0	0
	drift independent, $c_R^\mu = c_I^\mu = 0$			
	D: drift change, c_0^μ		E: threshold change, c_R^β	
	Horizon 1	Horizon 6	Horizon 1	Horizon 6
c_0^μ	2.3238	1.3771	2.3238	2.3238
c_R^μ	0	0	0	0
c_I^μ	0	0	0	0
c_0^β	0	0	0	0
c_I^β	0.0232	0.0232	0.0232	0.0138
c_R^β	-0.0387	0.1549	-0.0387	0.0918

the original model.

Next we fit the model to human behavior. As shown in Figure S5 the fit parameter values share several similarities to the original model. In particular, we see: a decrease in c_R^μ with horizon, consistent with a signal-to-noise ratio change driving random exploration; an increase in c_I^μ with horizon, consistent with an information bonus driving directed exploration; and a decrease in c_R^α with horizon.

In contrast to the original model we see no change in the baseline threshold with horizon c_0^β. Instead we see a significant change in the effect of reward on threshold (c_R^β) with horizon. In particular, c_R^β is positive for horizon 1 and approximately zero for horizon 6. This suggests that people increase their thresholds in horizon 1 when $|\Delta R|$ is high — that is, they make more careful decisions in horizon 1 when the consequences of making an error are largest.

While the modified model does not map directly onto the logistic model, both c_R^μ and c_R^β could affect behavioral variability and random exploration. To determine which of these factors contributes most to the horizon change in behavioral variability, we simulated behavior of the modified model in two conditions: first with c_R^β held constant with horizon (at its horizon 1 value) and second with c_R^μ held constant with horizon (at its horizon 1 value). We then fit the resulting behavior with the logistic choice model to estimate the effect of a horizon change in only one of c_R^β and c_R^μ on behavioral variability. As shown in Figure S6, we find that in both the [1 3] and [2 2] uncertainty conditions, the horizon change c_R^μ (i.e. when c_R^β is constant with horizon) accounts for most of the horizon change in the noise. Thus, these results with the modified model support the conclusion that random exploration is primarily driven by horizon changes in the signal-to-noise ratio, not by horizon change in threshold.

Finally, as with the original model, posterior predictive simulations show that the modified model provides a good fit to both the choice and response time data well.

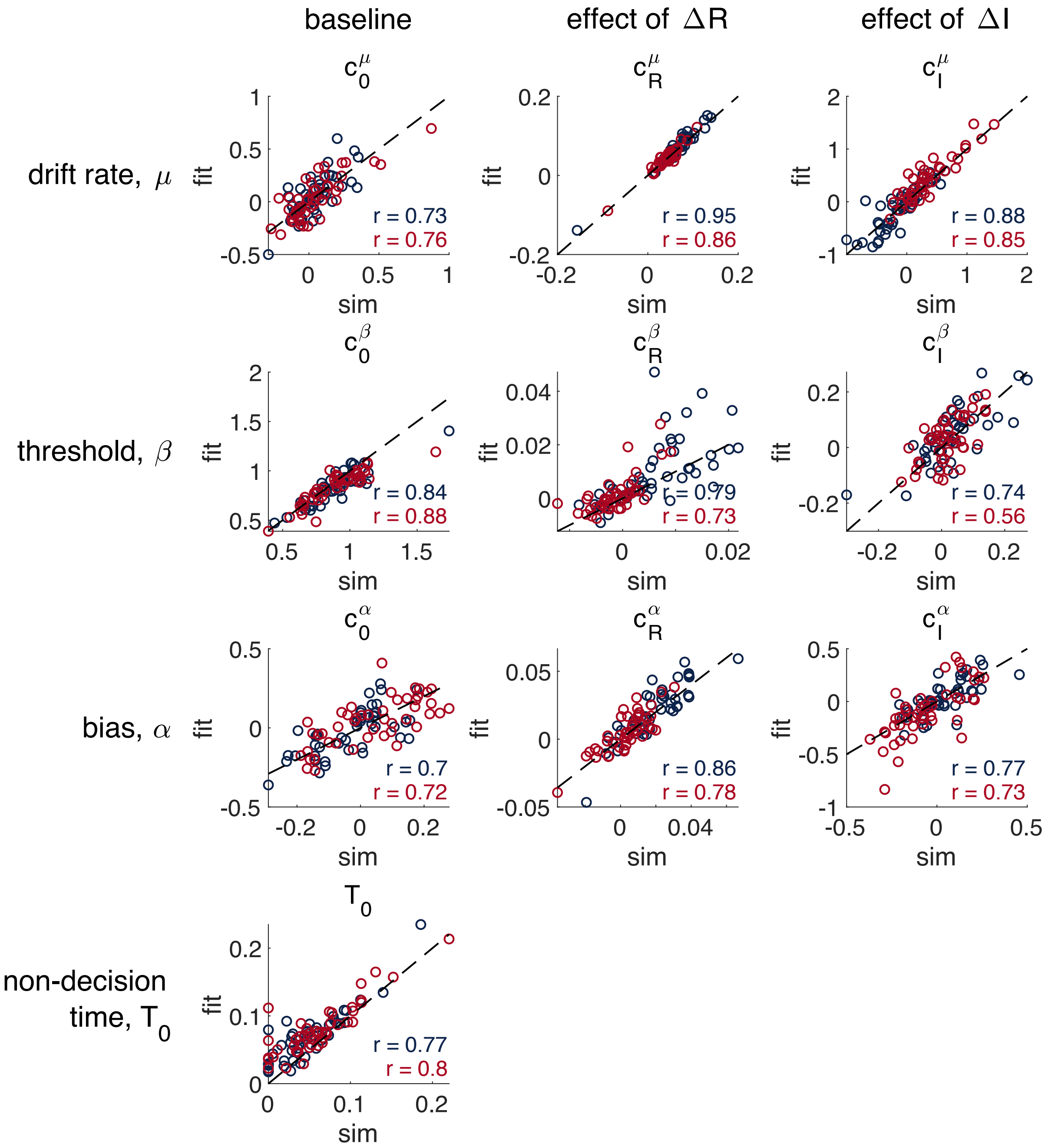

Figure S4: Parameter recovery in the modified model.

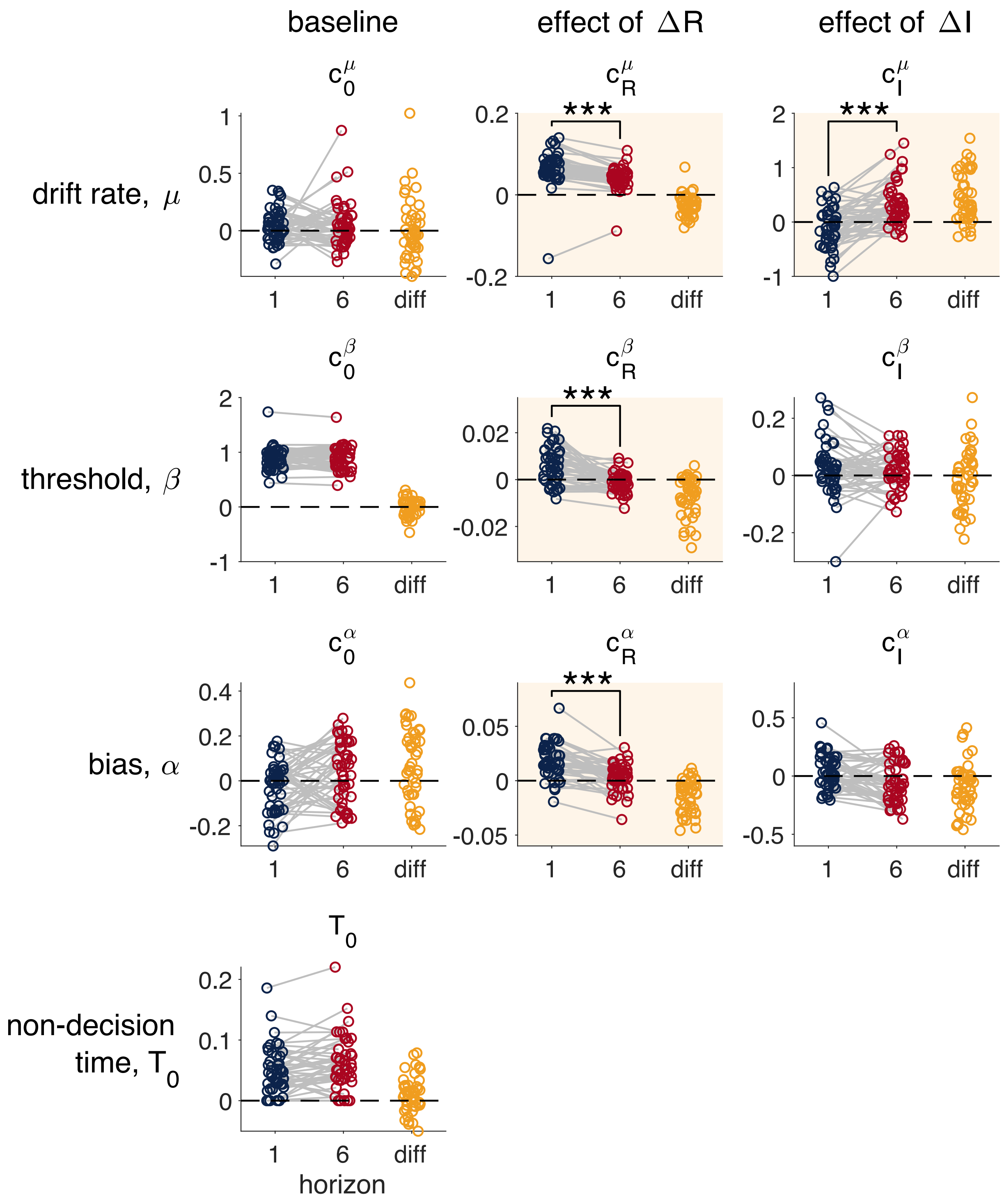

Figure S5: Fit parameter values for the modified model.

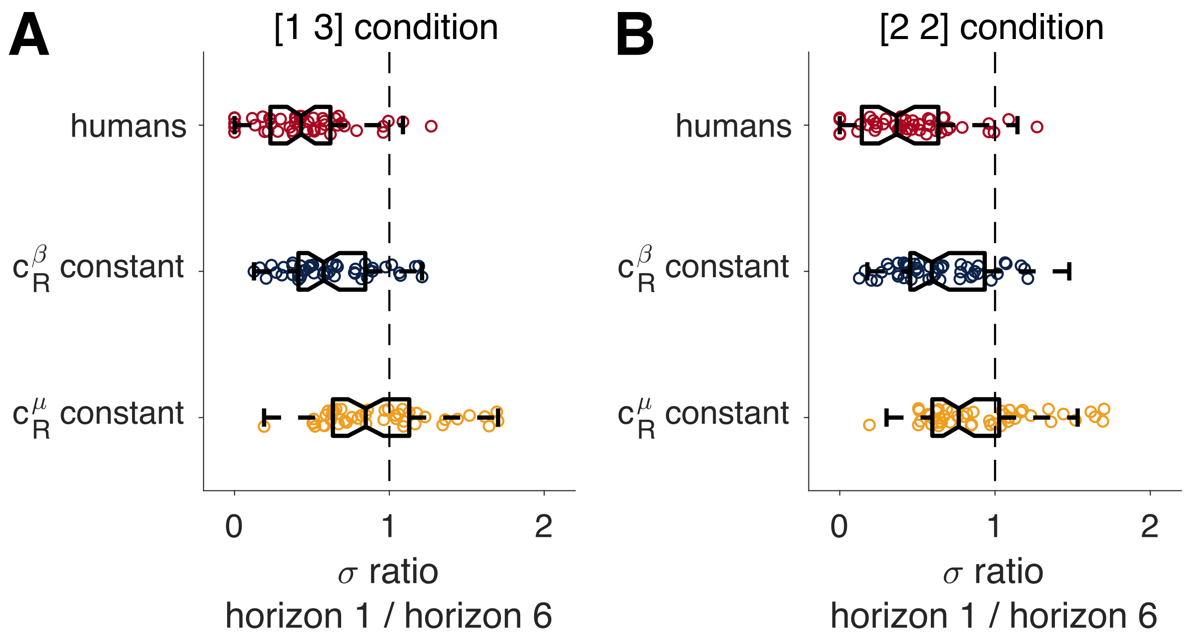

Figure S6: Sensitivity analysis for modified model

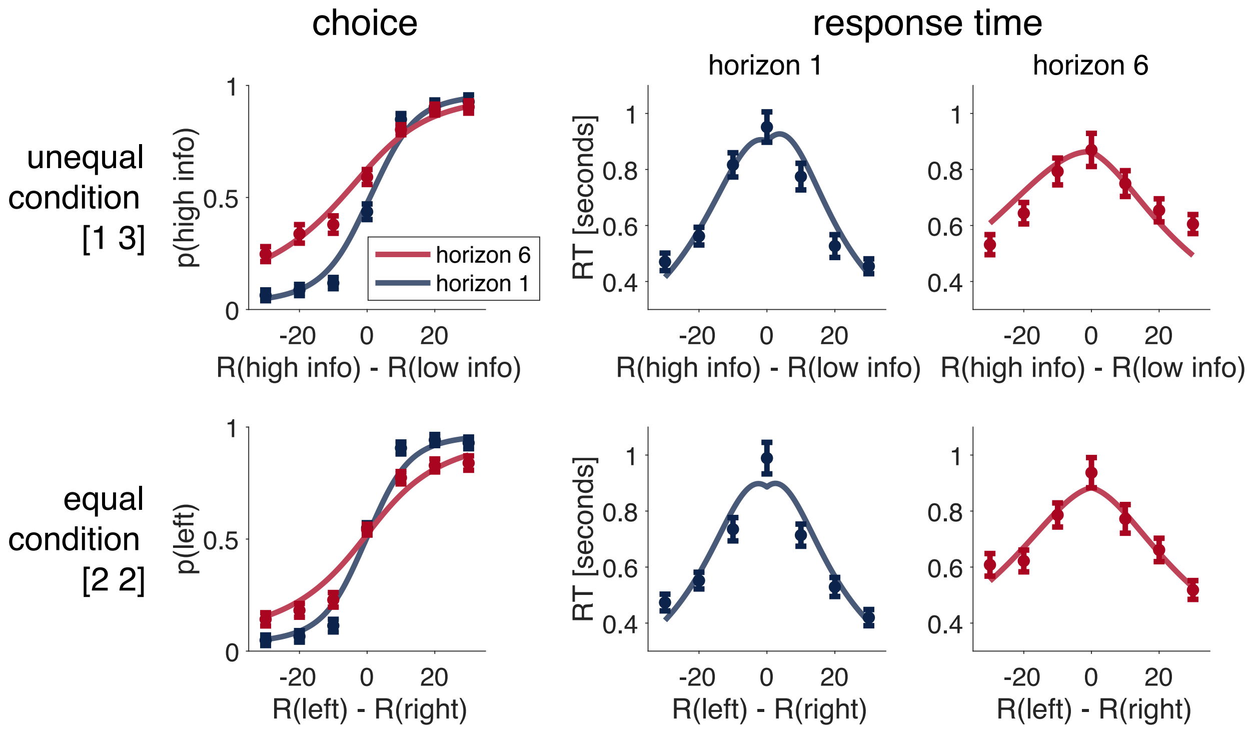

Figure S7: Posterior predictive for the modified model.
